# Case Report: Pasireotide treatment in neonatal congenital hyperinsulinism due to a homozygous *ABCC8* mutation

**DOI:** 10.3389/fendo.2026.1873953

**Published:** 2026-08-27

**Authors:** Calvin Kurz, Marcia Roeper, Lisa Friesl, Ertan Mayatepek, Thomas Meissner, Sebastian Kummer, Henrike Hoermann, Alena Welters

**Affiliations:** 1 Department of General Pediatrics, Neonatology and Pediatric Cardiology, Medical Faculty, University Hospital Düsseldorf, Heinrich-Heine-University, Düsseldorf, Germany; 2 Department of Neonatology and Pediatric Intensive Care, Children’s Hospital University of Bonn, Bonn, Germany

**Keywords:** congenital hyperinsulinism, infant, pancreatectomy, pasireotide, somatostatin analogue

## Abstract

Mutations in ABCC8 and KCNJ11 are associated with the most severe and diazoxide-resistant forms of congenital hyperinsulinism (CHI). Somatostatin analogues are commonly used off-label as second-line treatment. While octreotide and lanreotide are the most established options, pasireotide—a second-generation somatostatin analogue with higher affinity for somatostatin receptor subtype 5—has been hypothesized to offer improved suppression of insulin secretion. We report the off-label use of subcutaneous pasireotide in an infant with severe CHI due to a homozygous ABCC8 mutation. The patient presented with severe persistent hypoglycemia despite high-dose octreotide, continuous glucagon infusion, and high carbohydrate requirement. The established next step would have been a subtotal pancreatectomy. Following detailed counseling, the parents expressed a strong preference to defer surgical intervention and to pursue further medical options. Thus, an individualized therapeutic trial with subcutaneous pasireotide was initiated at approximately 8 weeks of age as an attempt to avoid surgery. However, pasireotide at doses of up to 0.11 mg/kg/day administered every 4 h did not lead to substantial clinical improvement. Instead, treatment was associated with increased glycemic variability, as reflected by more frequent episodes of hypo- and hyperglycemia, ultimately requiring reintroduction of glucagon as rescue therapy. No adverse effects were observed. Due to the lack of therapeutic response, pasireotide was discontinued after 8 days and the patient underwent near-total pancreatectomy. In conclusion, intermittent pasireotide injections were not associated with clinical improvement in this infant with medically refractory CHI, and its use potentially contributed to increased glycemic variability and instability. Further studies are needed to evaluate the safety and efficacy of pasireotide in this vulnerable patient population.

## 1 Introduction

Congenital hyperinsulinism (CHI) is a rare condition in which pancreatic β-cells inappropriately secrete insulin despite low blood glucose levels (1). It is the leading cause of recurrent and persistent hypoglycemia in newborns and children (1). The severity of CHI can vary significantly, ranging from mild to severe, depending on the underlying genetic cause (2).

To date, at least 30 genes have been described to be associated with CHI (3). Pathogenic variants in *ABCC8* and *KCNJ11* are associated with the most severe and treatment-resistant forms. These genes encode the two subunits of the ATP-sensitive potassium (K_ATP_) channel in pancreatic β-cells (2, 4).

The primary therapeutic goal is to prevent hypoglycemia and irreversible neurological damage, particularly in neonates and infants during critical phases of brain development (5). Treatment approaches are primarily dietary and pharmacological, aiming to maintain euglycemia through frequent feeding, carbohydrate supplementation, and pharmacological treatment (5).

Currently, diazoxide is the only approved medication for CHI (5). It acts by opening K_ATP_ channels to suppress insulin secretion and is used as first-line therapy. However, individuals with inactivating mutations in *ABCC8* or *KCNJ11* are often diazoxide-unresponsive due to malfunction of the K(_ATP_) channel (5).

In such cases, octreotide, a first-generation somatostatin analogue, is used off-label as second-line therapy (5, 6). It acts mainly via binding to somatostatin receptor subtype (SSTR) 2 and SSTR5 to inhibit insulin secretion (6). Additional off-label therapies—including long-acting somatostatin analogues, continuous glucagon infusion, sirolimus, nifedipine, and glucagon-like Peptide-1 (GLP-1) receptor antagonists—have been employed in selected severe cases. While long-acting somatostatin analogues represent an established alternative in patients responsive to somatostatin receptor-targeted therapy, the clinical utility of other approaches is limited by variable efficacy, adverse events, complex administration (e.g., glucagon), or restricted availability outside of specialized research settings (5). Thus, therapeutic options remain limited, and the lack of approved alternatives highlights the urgent need for new pharmacological agents for severe, medically refractory forms of CHI.

In patients for whom medical therapy does not sufficiently stabilize blood glucose levels, near-total pancreatectomy may become necessary. However, surgery is associated with significant risks, including lifelong pancreatic exocrine insufficiency and insulin-dependent diabetes mellitus. Furthermore, it is rarely curative: up to 60% of patients continue to experience persistent hypoglycemia after subtotal resection (1, 7). Accordingly, a conservative approach is generally recommended over surgical intervention, as disease severity may decline with age, potentially improving medical responsiveness over time.

In this context, pasireotide, a second-generation somatostatin analogue, has been proposed as a potential off-label alternative treatment for CHI. It binds to four of the five SSTRs, with particularly high affinity for SSTR1 and SSTR5 (8, 9). Pasireotide was first introduced for the treatment of Cushing’s disease and acromegaly and is known to cause hyperglycemia because of insulin inhibition (8). Based on its strong affinity for SSTR5, which is abundantly expressed in pancreatic β-cells, it has been considered a promising candidate for suppressing insulin hypersecretion in CHI patients unresponsive to conventional therapy. However, its clinical use in CHI remains off-label and limited to a few published case reports (4, 9, 10). Its clinical application is therefore primarily based on pharmacodynamic rationale and the hope of achieving improved glycemic control in patients unresponsive to standard treatment. Here, we present the case of a neonate with genetically confirmed CHI due to homozygous pathogenic variants in *ABCC8* and recurrent, severe hypoglycemia, who was treated with pasireotide as a compassionate off-label intervention during early infancy.

## 2 Case presentation

### 2.1 Initial presentation

The patient was born at 37 + 5 weeks of gestation via elective caesarean section in a peripheral hospital, as the fifth child of consanguineous Syrian-born parents. Birth weight was 4065 g (99th percentile, standard deviation score (SDS) +2.22). The pregnancy was complicated by insulin-dependent gestational diabetes; otherwise, it was uneventful.

Two siblings were diagnosed with diffuse CHI due to a homozygous nonsense mutation in the *ABCC8* gene and presented with severe neonatal hypoglycemia, recurrent seizures, structural epilepsy, and global developmental delay. Due to persistent, severe hypoglycemia despite medical therapy, both siblings underwent pancreatic surgery in Syria: one sibling received a near-total pancreatectomy at 3 months of age, while the other underwent two partial pancreatectomies at 4 and 11 months of age.

Given this family history, the newborn was admitted to the neonatal intensive care unit (NICU) immediately after birth for monitoring. Cardiorespiratory adaptation was normal.

The first postnatal blood glucose measurement at approximately 20 minutes of age revealed profound hypoglycemia (0.6 mmol/L (11 mg/dL)). A glucose bolus was administered intravenously, followed by early enteral feeding. Blood glucose rose transiently to 2.8 mmol/L (50 mg/dL). To stabilize glycemia, continuous intravenous glucose infusion (7 mg/kg/min) and 2-hourly oral feeds were initiated, resulting in a total carbohydrate intake of about 11.3 mg/kg/min.

Due to persistent hypoglycemia, glucose intake was gradually increased in the first days of life (up to 23.9 mg/kg/min). Hyperinsulinism was confirmed biochemically by detecting an inappropriately elevated insulin level of 312.5 pmol/L (reference: 12–96 pmol/L) during hypoglycemia (glucose < 2.8 mmol/L (50 mg/dL)).

The patient was transferred to our tertiary care center on the eighth day of life for further management of recurrent, severe hypoglycemia.

### 2.2 Clinical course after transfer

Upon admission to our center, management focused on optimizing medical and nutritional therapy to stabilize blood glucose levels. Continuous subcutaneous glucagon infusion (≈ 19.27 µg/kg/h; ≈ 2 mg/day) was initiated to support glycemic stability, and enteral nutrition was enriched with maltodextrin (total daily dose 1.8–2.2 g/kg/day). Following a normal cardiac evaluation, diazoxide therapy was started and rapidly titrated to a maximum dose of 15 mg/kg/day but discontinued thereafter due to lack of clinical response—consistent with diazoxide unresponsiveness expected from the presumed homozygous *ABCC8* mutation, which was inferred at this stage from the affected siblings’ phenotype.

**Figure 1:**
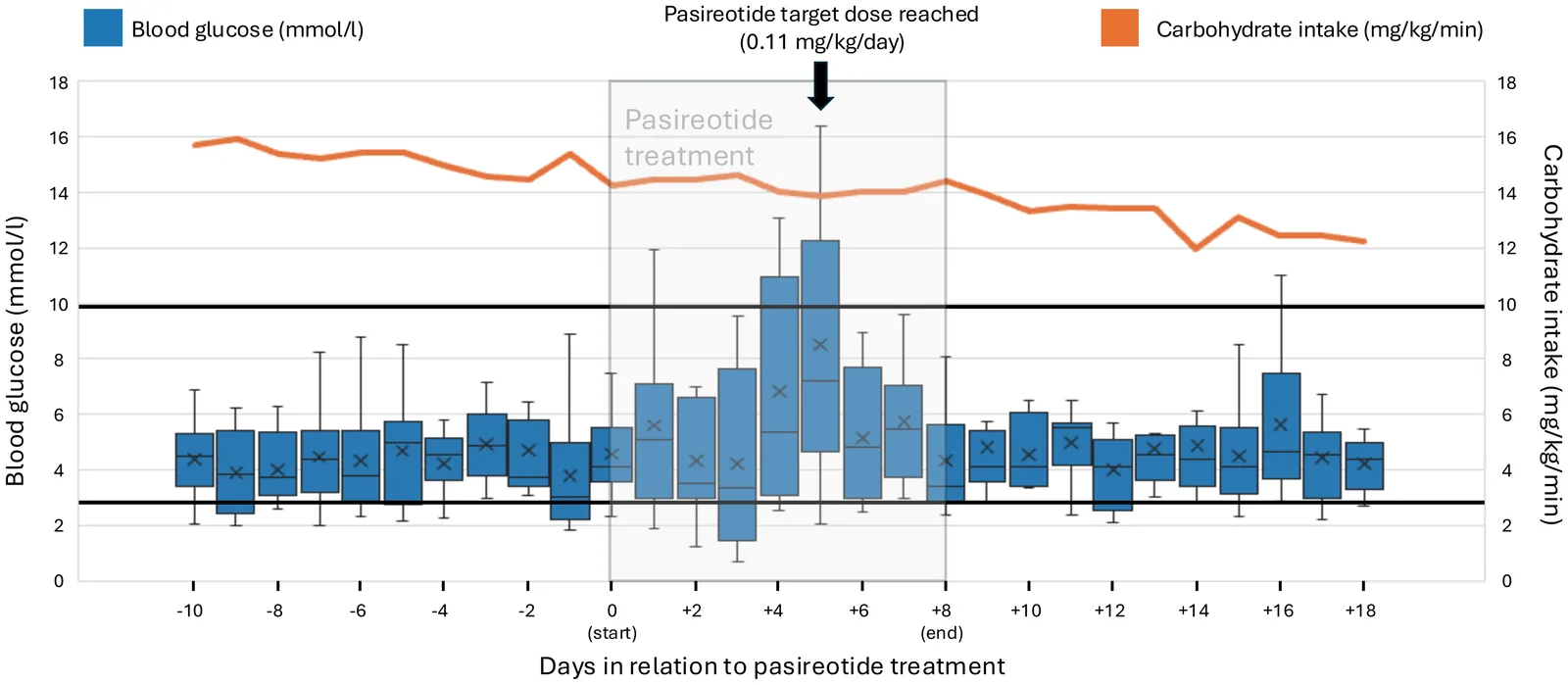
Mean daily blood glucose concentrations shown as boxplot (blue; median and interquartile range (IQR), with crosses [×] indicating the mean) and carbohydrate intake (orange line) during the period spanning 10 days before, during, and 10 days after pasireotide treatment. Pasireotide treatment and reaching of target dose are indicated by markers. No improvement in glycemic control was achieved during pasireotide treatment with increased glycemic variability. Carbohydrate intake remained elevated throughout the observation period.

Subcutaneous octreotide treatment was subsequently introduced with parental consent and increased up to 40 µg/kg/day in 3–4 daily injections. A trial of continuous subcutaneous infusion at the same cumulative daily dose did not further improve glycemic control. In parallel, intensive nutritional support was required, including continuous enteral glucose infusion via a feeding tube (max. carbohydrate intake 15.6 mg/kg/min) and later gastrostomy placement due to severe oral aversion. Despite these measures, glycemic stability could not be achieved. **Figure 1** provides an integrated overview of the patient’s enteral and pharmacologic treatment in relation to glycemic trends throughout the clinical course.

Despite maximal medical treatment and exhaustive enteral nutritional support – providing approximately 15 mg/kg/min carbohydrates (8.5 mg/kg/min via regular feeds enriched with maltodextrin to a total carbohydrate concentration of approximately 8.9% and 6.5 mg/kg/min via continuous dextrose tube feeding) – the child continued to experience severe, unpredictable hypoglycemic episodes. Therefore, prior to the next therapeutic step, pancreatic surgery, off-label use of pasireotide—a second-generation somatostatin analogue with higher affinity for SSTR5—was initiated at the age of approximately 2 months; however, it did not result in improved glycemic control, necessitating reintroduction of glucagon as rescue therapy during ongoing pasireotide treatment (see below for details).

Because of persistent metabolic instability, glucagon dependency, excessive weight gain, and severe oral aversion, surgical intervention was considered unavoidable. After interdisciplinary consultation, a near-total (85–90%) pancreatectomy was performed 18 days following discontinuation of pasireotide treatment. Postoperatively, glycemic control improved markedly, allowing discontinuation of glucagon infusion and continuous glucose feeding. Octreotide was tapered to 13.3 µg/kg/day, and pancreatic enzyme replacement treatment was initiated due to exocrine insufficiency. The patient was discharged in good overall condition at the age of approximately 3.5 months, with stable glycemia, maintained on subcutaneous octreotide 40 µg three times daily (13.3 µg/kg/day) and a structured enteral feeding regimen of eight feeds per day (110 mL each; ≈ 97.5 mL/kg/day).

### 2.3 Genetic diagnosis

Genetic diagnostics initiated shortly after birth identified the suspected pathogenic homozygous *ABCC8* variant c.4612C>T (p.Arg1538*), which was also present in the patient’s siblings. This nonsense variant introduces a premature stop codon resulting in truncation of the SUR1 protein. It is classified as pathogenic/likely pathogenic in ClinVar. To our knowledge, this specific variant has not previously been reported in a patient with CHI. Notably, a different heterozygous ABCC8 variant affecting the same amino acid residue, c.4613G>A (p.Arg1538Gln), was recently reported in a patient with maturity-onset diabetes of the young (MODY); this variant had previously been associated with CHI, illustrating that different substitutions at the same codon may be associated with distinct phenotypes (11).

## 3 Focus-section: pasireotide-trial

In the setting of persistent severe hypoglycemia despite maximal medical management with high-dose octreotide, continuous subcutaneous glucagon infusion, and intensive nutritional support, no further established pharmacologic options were available, and it was not possible to discharge the patient home. Alternative pharmacological options were considered but not pursued. Given its potential for serious adverse effects in infancy, sirolimus was not considered a suitable therapeutic option (12, 13). Long-acting somatostatin analogues (e.g., lanreotide) were likewise not selected, because the patient was considered too young for a long-acting formulation. Given the risk of serious adverse events of somatostatin analogues in early infancy (particularly necrotizing enterocolitis), we favored a short-acting agent that allowed immediate treatment discontinuation if necessary (14). Therefore, off-label therapeutic use of pasireotide, a second-generation somatostatin analogue, was initiated after intensive discussion and joint decision with the parents. This decision was based on its distinct receptor-binding profile: while octreotide predominantly targets SSTR 2 and 5, pasireotide binds with high affinity to SSTR1, SSTR2, SSTR3, and particularly SSTR5 (8). Since SSTR5 is highly expressed on pancreatic β-cells and plays a key role in the suppression of insulin secretion, pasireotide was hypothesized to offer superior glycemic control in cases unresponsive to first-generation analogues (4, 8, 9).

Pasireotide was initiated with 0.075 mg subcutaneously twice daily (0.019 mg/kg/day) and was rapidly escalated in response to persistent dysglycemia: first to 0.22 mg three times daily (0.083 mg/kg/day) and subsequently adjusted to 0.15 mg six times daily (0.11 mg/kg/day) to reduce glycemic excursions by shortening the dosing intervals. However, neither the initiation of treatment with pasireotide nor the intensification of the daily dose and distribution across multiple injections led to an improvement in glycemic stability. Instead, continuous subcutaneous glucagon infusion, which had initially been paused, needed to be resumed during ongoing pasireotide treatment due to insufficient glycemic control with recurrent hypoglycemic episodes (see **Figure 1**).

**Figure 2:**
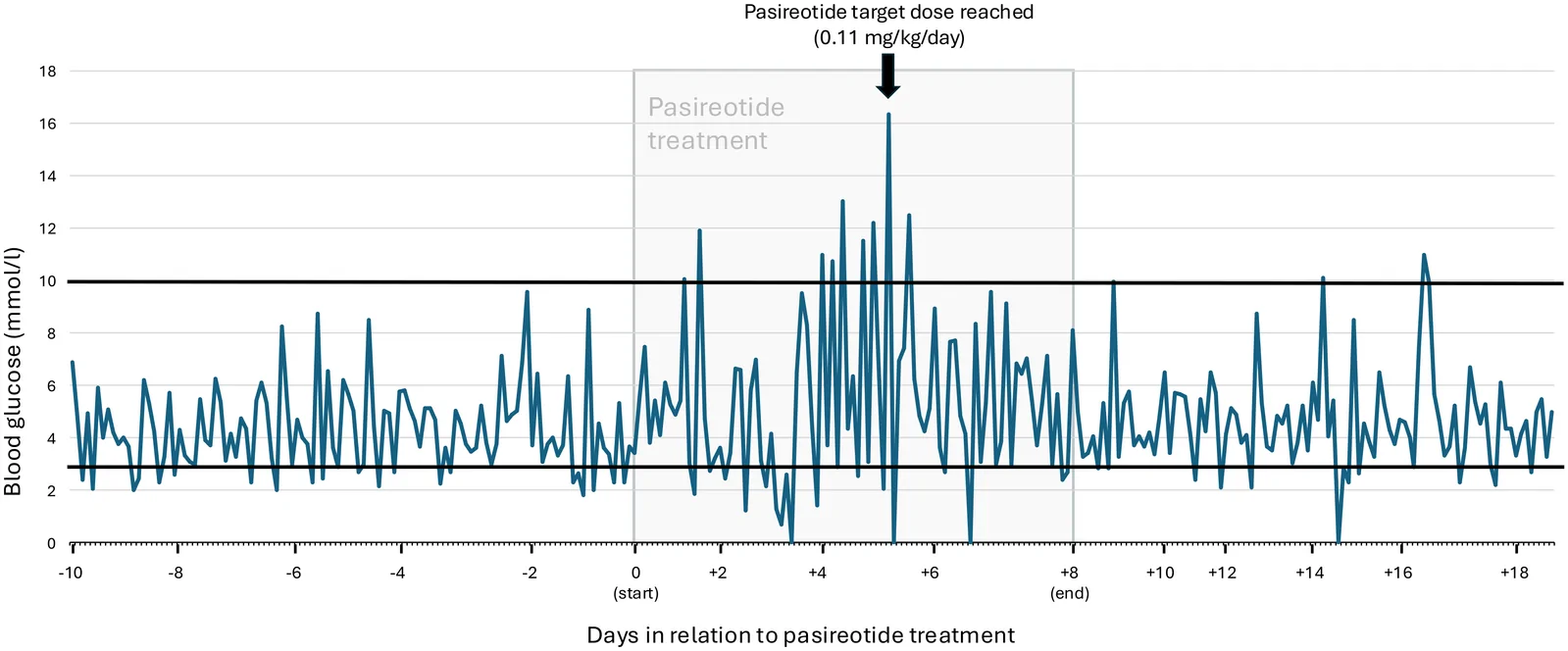
Individual blood glucose measurements over time from 10 days prior to pasireotide initiation until 10 days after its discontinuation. Pasireotide treatment period and reaching of target dose are indicated by markers. The period of pasireotide use is associated with increased glycemic variability, with more frequent and pronounced excursions into both hypoglycemic and hyperglycemic ranges.

Precisely, following the initiation of pasireotide treatment and subsequent dose escalation, we observed an increasing glycemic variability. Although mean blood glucose concentrations remained in a similar range across all three phases (pre-, during, and post-pasireotide), broad fluctuations with frequent shifts between hypo- and hyperglycemia were observed during the period of pasireotide administration. Fluctuations emerged early after treatment initiation and became more pronounced with dose escalation (**Figures 1**, **2**).

**Figure 3:**
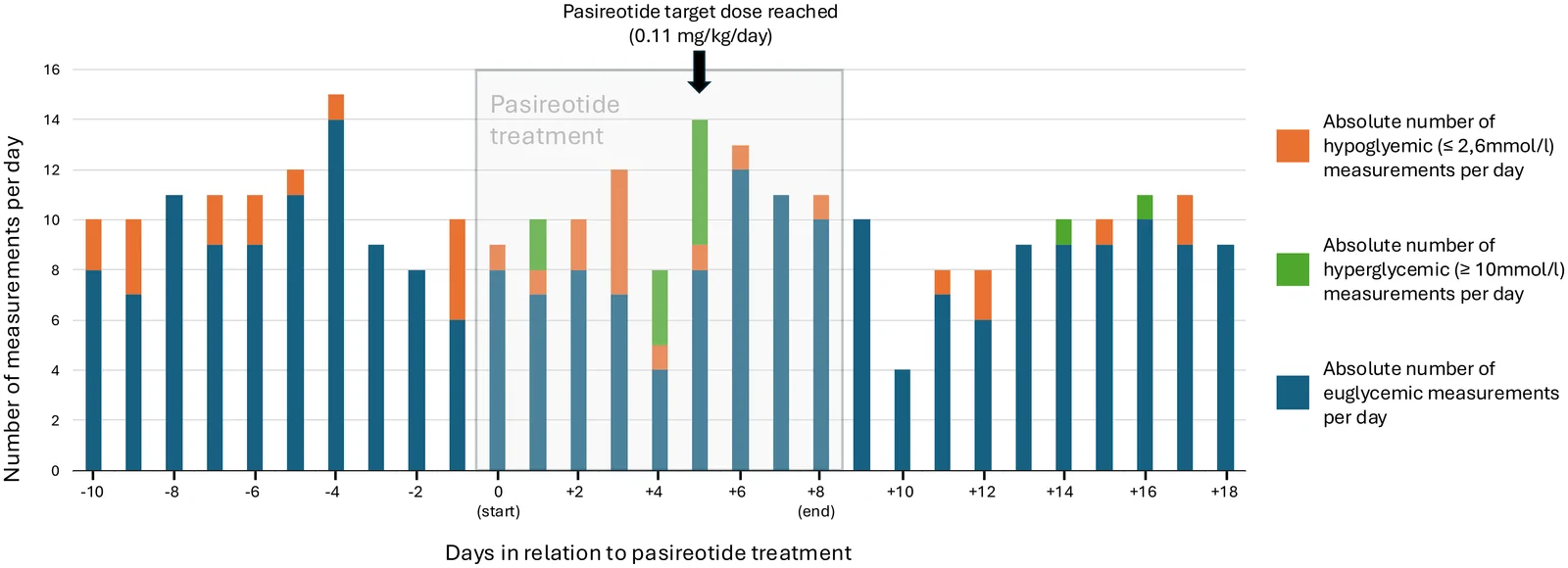
Daily count of hypoglycemic (orange), euglycemic (blue), and hyperglycemic (green) blood glucose readings during the same observation period. Pasireotide treatment period and reaching of target dose are indicated by markers. During pasireotide treatment, there is a marked increase in both hypoglycemic and hyperglycemic measurements, while the number of euglycemic values (defined as glucose levels between 2.8–10 mmol/L) declines. These findings indicate a deterioration of glycemic stability under pasireotide treatment.

**Figure 3** further illustrates the increase in glycemic variability observed during pasireotide treatment, showing a concurrent rise in both hypo- and hyperglycemic episodes and a reduction in euglycemic readings. These findings indicate that multiple daily pasireotide injections, despite its broader receptor activity, did not achieve the anticipated glycemic stabilization in this patient.

Given the absence of clinical benefit, pasireotide was discontinued, and octreotide therapy was resumed. No adverse effects due to pasireotide were observed during the short treatment course.

## 4 Discussion

Pasireotide, a second-generation somatostatin analogue, has been considered an off-label therapeutic option for CHI, particularly in patients who fail to respond to standard medical treatments. Its higher affinity for SSTR5 —in contrast to octreotide’s predominant action via SSTR2—provided a mechanistic rationale for use in this case of severe, diazoxide-unresponsive CHI caused by a homozygous *ABCC8* mutation. Alternative agents such as sirolimus and long-acting somatostatin analogues were considered but not pursued, primarily because of concerns regarding their safety profile in this young infant with severe CHI (12–14).

Despite this theoretical advantage, pasireotide did not improve glycemic control in our patient, even after gradual dose escalation and redistribution of dosing from 0.075 mg twice daily (0.019 mg/kg/day) to 0.15 mg six times daily (0.11 mg/kg/day). Instead, the treatment was associated with increased glycemic variability, accompanied by a notable rise in the number of dysglycemic episodes (**Figure 3**). Whether this reflected greater pharmacodynamic effects of pasireotide, the intermittent mode of subcutaneous administration, concurrent therapeutic changes, or a combination of these factors cannot be determined from this single case. No pharmacokinetic or pharmacodynamic data were obtained, and any mechanistic explanation therefore remains speculative. However, under this regimen, the observed net change was a deterioration of glycemic stability rather than a therapeutic benefit.

This clinical course is in line with the findings reported by Mooij et al. (2021), where pasireotide was trialed in a child with CHI following partial pancreatectomy (4). While some glycemic response was observed, pronounced fluctuations in blood glucose persisted throughout the treatment, raising concerns about the reliability of pasireotide as a stabilizing agent. Our findings mirror that experience and support the notion that pasireotide may, in some cases, exacerbate rather than stabilize glycemic variability.

By contrast, Telehuz et al. (2024) reported a more favorable outcome in twin neonates with CHI, where pasireotide contributed to better glycemic control and avoided the need for surgery (10). However, several important differences between their report and our case must be considered. Most importantly, in the study by Telehuz et al., pasireotide was administered as a continuous subcutaneous infusion over an extended period during the first year of life, whereas in our patient, therapy was given intermittently and discontinued after a short trial due to lack of efficacy. The dosing regimen also differed markedly, with a lower cumulative daily dose in the twins (max. 1200 µg/m²/day vs. max. 2647 µg/m²/day in our case). After 12 months, treatment of the twins was transitioned to long-acting pasireotide with injections every four weeks, and diazoxide was reintroduced—making the specific contribution of pasireotide to the observed glycemic stability uncertain. We consider the mode of administration – continuous infusion versus repeated bolus injections – to be a plausible explanation of the differing clinical observations. Continuous infusion would be expected to provide more stable drug exposure than intermittent dosing, although this was not assessed in our patient. We therefore hypothesize that intermittent administration may have contributed to the observed glycemic variability. Consequently, our findings do not permit firm conclusions regarding the intrinsic efficacy of pasireotide in CHI; they may instead reflect the mode of drug delivery rather than the pharmacological properties of pasireotide itself. This distinction could not be resolved in our case because continuous infusion was not attempted and warrants further investigation.

Although all patients carried pathogenic *ABCC8* variants predicted to result in diffuse, diazoxide-unresponsive CHI, differences in mutation type, together with marked differences in dosing strategy, treatment duration, and mode of administration, may have contributed to the divergent therapeutic outcomes. Finally, nutritional management also differed, as our patient required substantially higher carbohydrate intake to maintain euglycemia compared with the cases described by Telehuz et al., further underlining the heterogeneity of metabolic control and treatment response among patients with severe *ABCC8*-related CHI.

Insights from other clinical settings further inform the discussion of pasireotide’s role in hyperinsulinemic conditions. In a case report by Oziel-Taieb et al. (2022), pasireotide was successfully used to manage refractory hypoglycemia in a patient with malignant insulinoma. In that adult patient, pasireotide proved effective after failure of conventional therapy (9). While not directly comparable to CHI in neonates, this case illustrates the broader applicability of pasireotide in insulin-mediated hypoglycemic disorders—and highlights the contrast between its potential utility in adult neuroendocrine tumors versus congenital forms of dysregulated insulin secretion.

It is also important to consider the safety profile of pasireotide. Although no adverse effects occurred during our short treatment trial, long-term use is associated with risks such as adrenal suppression, gastrointestinal symptoms, hyperglycemia, and elevated liver enzymes (8). In our patient, safety monitoring during pasireotide treatment was limited to liver enzymes and clinical assessment of gastrointestinal symptoms and growth parameters; adrenal-axis function was not systematically assessed. No abnormalities were noted in the monitored parameters during the short treatment course. These risks must be carefully weighed, particularly in vulnerable neonatal populations, and underscore the need for close, structured monitoring during off-label use.

A key strength of this case is the detailed and regular documentation of blood glucose values before, during, and after pasireotide treatment. This high-frequency monitoring provides a robust picture of therapeutic response and glycemic variability.

Several limitations must be acknowledged. First, as this report describes a single-patient undergoing concurrent therapeutic changes, including the reintroduction of glucagon, the observed changes during pasireotide treatment should be interpreted as associations rather than evidence of a causal effect, and the findings cannot be generalized beyond this individual case. Second, glycemic variability was assessed qualitatively rather than with formal metrics such as coefficient of variation or time in range. Although such metrics facilitate comparisons across studies, they are primarily intended for continuous glucose monitoring (CGM) data obtained at fixed sampling intervals. In contrast, our patient underwent intermittent point-of-care glucose measurements, which provide greater analytical accuracy than CGM but were performed at variable, clinically indicated time points, precluding meaningful calculation of these metrics. We therefore chose to present the raw glycemic data transparently (**Figures 1**, **2**) rather than derive quantitative summary metrics that could imply a level of precision not supported by the underlying data. Third, the treatment duration was short, and a delayed therapeutic effect cannot be excluded. Finally, no pharmacokinetic or pharmacodynamic data were available; therefore, the potential influence of intermittent dosing on the observed glycemic variability remains hypothetical.

Taken together, our findings—alongside the heterogenous outcomes reported in the literature—underscore the inconsistent clinical utility of pasireotide in CHI. While selected cases may benefit from its unique receptor affinity, responses are variable and may be influenced by dosage and mode of administration, as well as genetic, metabolic, and contextual factors. Further controlled studies are needed to define the role of pasireotide in CHI, including the optimal mode of administration, and to identify patient profiles more likely to respond favorably.

## 5 Conclusions

In this infant with severe, genetically confirmed congenital hyperinsulinism due to a homozygous *ABCC8* mutation, off-label pasireotide administered by intermittent subcutaneous injection was not associated with improved glycemic control despite dose escalation, and ultimately could not prevent near-total pancreatectomy. Instead, treatment was associated with increased glycemic variability, ultimately necessitating the reintroduction of glucagon therapy. Taken together with previous reports, our findings raise the possibility that differences in the mode of administration may partly account for the heterogenous clinical responses reported in CHI. Specifically, intermittent subcutaneous administration may be insufficient to achieve stable glycemic control in severe neonatal CHI, as suggested by the limited published evidence. Whether continuous subcutaneous infusion offers advantages over intermittent administration warrants further evaluation.

This report highlights the importance of detailed glycemic monitoring in off-label therapies for this rare disease and calls for the development of standardized protocols and further studies to better define the role, safety, and efficacy of pasireotide in CHI.

## Data Availability

The original contributions presented in the study are included in the article/supplementary material. Further inquiries can be directed to the corresponding author.
